# Respiratory Distress in the Newborn with Primary Ciliary Dyskinesia

**DOI:** 10.3390/children8020153

**Published:** 2021-02-18

**Authors:** Evans Machogu, Benjamin Gaston

**Affiliations:** Section of Pediatric Pulmonology, Allergy and Sleep Medicine, Indiana University School of Medicine, Riley Hospital for Children, Indianapolis, IN 46202, USA; begaston@iu.edu

**Keywords:** primary ciliary dyskinesia, neonatal respiratory distress

## Abstract

Primary ciliary dyskinesia (PCD) is inherited in a predominantly autosomal recessive manner with over 45 currently identified causative genes. It is a clinically heterogeneous disorder that results in a chronic wet cough and drainage from the paranasal sinuses, chronic otitis media with hearing impairment as well as male infertility. Approximately 50% of patients have situs inversus totalis. Prior to the development of chronic oto-sino-pulmonary symptoms, neonatal respiratory distress occurs in more than 80% of patients as a result of impaired mucociliary clearance and mucus impaction causing atelectasis and lobar collapse. Diagnosis is often delayed due to overlapping symptoms with other causes of neonatal respiratory distress. A work up for PCD should be initiated in the newborn with compatible clinical features, especially those with respiratory distress, consistent radiographic findings or persistent oxygen requirement and/or organ laterality defects

## 1. Neonatal Respiratory Distress

Respiratory distress develops in about 7% of all newborn infant deliveries [1]. Respiratory distress syndrome (RDS) develops in 1% of all newborn infant deliveries primarily in premature babies due to surfactant deficiency, and its prevalence is inversely associated with gestational age. More than 50% of infants born at <28 weeks’ gestation develop RDS compared to <5% of those greater than 37 weeks gestation. In 2014, nearly 10 of every 100 infants were delivered at <37 completed weeks’ gestation. Neonatal RDS contributes to significant morbidity and mortality and is ranked in the top 10 leading causes of infant mortality. It is the leading cause of mortality in premature infants and contributes to 2% of all causes of infant deaths [2].

Clinical symptoms of respiratory distress in neonates include tachypnea, nasal flaring, grunting, retractions and cyanosis. The differential diagnosis for neonatal respiratory distress is broad and includes RDS, transient tachypnoea of the newborn (TTN), neonatal pneumonia, pulmonary air leak such as pneumothorax and pneumomediastinum, pulmonary arterial hypertension, congenital heart disease, interstitial lung diseases such as those due to surfactant protein deficiencies and other non-pulmonary systemic disorders such as neonatal sepsis, hypothermia and metabolic acidosis. Primary ciliary dyskinesia (PCD) presents with neonatal respiratory distress in majority of patients and should be considered in the differential diagnosis, especially in term infants with respiratory distress. Given the long-term complications related to chronic airway inflammation and infection, making the diagnosis in the neonatal period is critical.

## 2. The Motile Cilia

Primary ciliary dyskinesia is characterized by impaired function of motile cilia that leads to chronic inflammation and infection in the middle ear, sinuses and lungs among other clinical manifestations. Motile cilia are highly specialized organelles that exist in select cells in the upper and lower respiratory epithelium, the ependymal cells in the cerebral ventricles, in the female reproductive tract and in spermatozoa.

Each motile cilium consists of a cytoskeleton referred to as an axoneme that is composed of nine longitudinal microtubule doublets that surround a central pair of single microtubules in a 9+2 axonemal conformation. The outer microtubules are linked to each other through nexin links and have two attachments, the inner and outer dynein arms (IDA and ODA). The dynein arms are anchored to the central microtubules via radial spokes [3]. The cilium is attached to the cell via a basal body and, in the airway lumen, extends through a periciliary liquid (PCL) layer with the tip reaching a mucus layer. Each ciliated cell in the respiratory epithelium has approximately 200 cilia that beat at a frequency of 5–20 hz. The coordinated and synchronized bending of the cilia produces wave like movements that function to propel bacteria and trapped particles on the mucus layer of the airway surface [4]. Nodal cilia are also motile cilia that only exist in embryogenesis. Nodal cilia lack radial spokes and central microtubules and therefore have a 9+0 axonemal conformation. The outer microtubules are linked to each other and do have dynein arms. These cilia have a rotary motion that through chemical gradients help guide organ growth and laterality.

**Figure 1:**
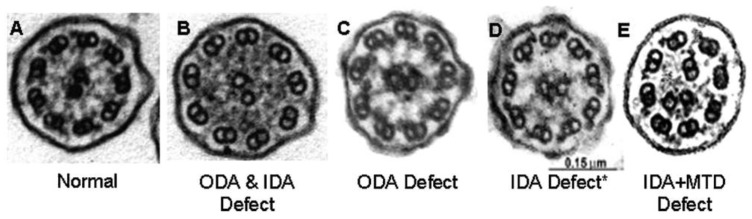
Ciliary defects as seen on transmission electron microscopy. Legend: Electron microscopy findings in primary ciliary dyskinesia. Diagnostic ciliary electron microscopy findings in primary ciliary dyskinesia. Normal ciliary ultrastructure (**A**), Outer and inner dynein arm defect (**B**), Outer dynein arm defect (**C**), Inner dynein arm defect alone (**D**), Inner dynein arm defect with microtubule disorganization (**E**). * Inner dynein arm defects alone are quite rare as a cause of PCD and usually due to secondary artifact. ODA: Outer Dynein Arm, IDA: Inner Dynein Arm, MTD: Microtubular Disorganizatio. Reprinted with permission from Shapiro et al. [8].

Approximately 70% of patients with PCD have biallelic mutations in a known gene associated with the disease. Mutations in these genes that code for the proteins that make up the ciliary axoneme result in ultrastructural and/or functional abnormalities of the motile cilia resulting in the PCD disorder [5]. However, many of the disease-causing genes do not result from genes encoding ciliary ultrastructure: additional genes are involved with ciliagenesis, trafficking of ciliary proteins, ciliary function and other cellular mechanisms not evident by electron microscopy. These ultrastructural defects can be visualized on transmission electron microscopy (TEM) and include ODA defects, ODA+IDA defects, IDA with microtubular disorganization (MTD), radial spoke defects or central apparatus defects (Figure 1). However, not all gene mutations result in obvious ultrastructural defects on TEM. In fact, approximately a third do not. For example, normal ciliary ultrastructure is found in mutations of the CCDC65 protein [6] while mutations in genes such as GAS8 result in very subtle changes that are not easily recognizable on TEM [7]. Further, because of chronic inflammation and sampling issues, TEM is falsely positive in up to a third of subjects. These issues have made TEM often unsuitable to rule in or out PCD, except in certain unequivocal cases.

## 3. Primary Ciliary Dyskinesia

Primary ciliary dyskinesia is caused by biallelic pathogenic mutations in the approximately 70% of patients with an identified PCD causative gene. The disorder was described by Kartagener in 1933 as a clinical triad of chronic sinusitis, bronchiectasis and situs inversus [9]. In 1975, Afzelius et al. demonstrated ultrastructural abnormalities in the dynein arms of the cilium that resulted in lack of cilia motility and infertility associated with recurrent sinopulmonary infections [10,11]. He named this the “immotile cilia syndrome”. Development of high-speed video microscopy further showed that functional ciliary impairment without apparent ultrastructural deformities, as well as motile cilia with abnormal movement patterns, could result in clinical disease [12,13]. The syndrome was therefore renamed primary ciliary dyskinesia [14].

Primary ciliary dyskinesia occurs in approximately 1 in 15,000 to 1 in 20,000 individuals [15] but is often underdiagnosed. Patients with situs inversus are more likely to be diagnosed at an earlier age, but diagnosis in general is often delayed to a mean age 4.4–6 years, after several years of symptoms [16,17]. This delay is likely due to low index of suspicion in the context of overlapping symptoms with other chronic respiratory diseases with a similar clinical presentation such as cystic fibrosis (CF), chronic aspiration, asthma, immunodeficiency, allergic rhinitis and protracted bacterial bronchitis.

**Figure 2:**
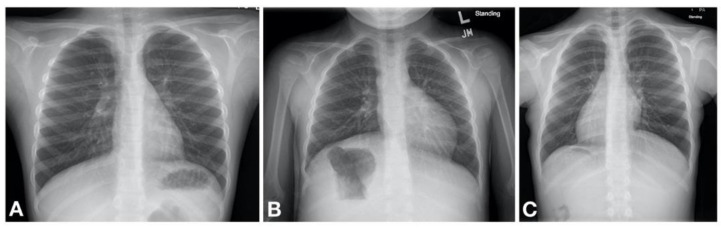
Machogu et al. Laterality defects found on chest radiography of patients with PCD. (**A**) Normal; Left sided cardiac apex with left sided stomach and right sided liver (**B**) Situs Ambiguous; Left sided cardiac apex with right sided stomach and left sided liver (**C**) Situs Inversus Totalis; right sided cardiac apex with right sided stomach and left sided liver.

The abnormal function of motile cilia results in impaired mucociliary clearance from the respiratory system and stagnation of purulent mucus that inevitably leads to recurrent and chronic oto-sino-pulmonary infections [18]. The PCD clinical phenotype is heterogenous and disease progression is likely related to the associated gene mutations [19]. Nonetheless, nearly all patients with PCD have early onset, year round wet and productive cough, while about 80% have early onset, year round daily nasal congestion and chronic sinusitis [20]. Recurrent bronchitis and pneumonia ultimately leads to development of bronchiectasis [21]. Additional clinical features include situs inversus totalis (SIT) in about half of the patients [18] while about 12% are reported to have other forms of heterotaxy [22] (Figure 2). Infertility is present in nearly all male patients due to reduced motility of spermatozoa [18]. Table 1 summarizes the clinical manifestations of PCD.

## 4. Respiratory Distress in the Newborn with Primary Ciliary Dyskinesia

Primary ciliary dyskinesia typically causes respiratory distress in the neonate with more than 80% of patients presenting with symptoms within the first 1–2 days of life [19,23,24,25,26,27]. It is thought that the impaired mucociliary clearance in the newborn results in atelectasis and lobar collapse due to mucus impaction which are notable on chest radiography in neonates with PCD developing respiratory distress [23]. These initial clinical manifestations are often transient, although some patients may have persistent oxygen requirement lasting weeks to months. Gradually a daily wet cough as well as nasal congestion with drainage become noticeable [19]. It is therefore likely that the initial transient nature of the symptoms poses a missed opportunity for diagnosis due to a low index of suspicion for PCD to initiate a workup. Some helpful distinguishing features between PCD and other causes of neonatal respiratory distress include the following. First, there is a somewhat later onset of symptoms; infants with PCD may not develop respiratory distress until 12–24 h after birth [23]. Second, the patient is typically a term infant without other obvious risk factors for respiratory distress. Third, the chest imaging often shows lobar atelectasis, rather than diffuse changes characteristic of, for example, TTN; although this is not universal. Fourth, many PCD patients have situs inversus totalis (approximately 50% of patients) [18] or other forms of heterotaxy (12% of patients). Therefore, a work up for PCD should be concurrently initiated in the neonate with respiratory distress especially in those with compatible clinical and/or radiological features.

## 5. Diagnosis of Primary Ciliary Dyskinesia

In spite of unexplained neonatal respiratory distress in more than 80% of neonates with PCD [23] the diagnosis of PCD is often delayed [16]. Varying ciliary structure and function results in varying times of manifestation of the classic PCD phenotype [8] and may contribute to the delay in diagnosis. There is not a “Gold standard” diagnostic test for PCD and a panel of diagnostic tests is therefore recommended to support the diagnosis with a greater number of positive tests resulting in a higher likelihood of having PCD in those meeting clinical criteria [8]. It is important to note that several similarities and differences exist between the recommended tests per the guidelines put forth by the European respiratory society (ERS) in 2017 [28] and the American thoracic society (ATS) in 2018 [29]. These differences are clearly elucidated in an editorial by Shoemark et al. [30] These tests include; (1) PCD genetic test panels; and (2) ciliary biopsy or brush biopsy culture with TEM. Note that immunofluorescence techniques are available in many European centers and will likely become, increasingly, a useful tool in the neonate. For older patients (over five years) nasal nitric oxide measurement can be successfully completed, but this is not suitable for use in newborns (3) additional tests not currently recommended in the ATS diagnostic panel include cytologic analysis or ciliary motion, which can be falsely negative [29]. These tests are, however, recommended by the ERS when used in conjunction with other PCD tests.

The suggested criteria for the diagnosis of PCD by age are summarized in the 2016 PCD foundation consensus statement [8]. Note that the utility of some of the diagnostic tests are further clarified in the 2018 guidelines [29]. After excluding other diseases with overlapping clinical symptoms, the major criteria include;

1. Unexplained neonatal respiratory distress (at term birth) with lobar collapse and/or need for respiratory support with continuous positive airway pressure (CPAP) and/or oxygen for >24 h.
2. Any organ laterality defect—situs inversus totalis, situs ambiguous, or heterotaxy.
3. Daily, year-round wet cough starting in first year of life or bronchiectasis on chest CT.
4. Daily, year-round nasal congestion starting in first year of life or pansinusitis on sinus CT.

Leigh et al. reported a specificity for diagnosis of PCD in early childhood of greater than 96% in patients presenting with a combination 3 or more of the major criteria [26]. In the neonatal period, a diagnosis of PCD can be made based on a combination of unexplained respiratory distress in a term birth and any organ laterality defect (including situs inversus totalis, situs ambiguous or heterotaxy) plus at least a diagnostic ciliary ultrastructure on TEM or presence of biallelic mutations in one PCD-associated gene [8]. High speed video microscopy or ciliary beat frequency or waveform analysis are no longer suggested initial diagnostic tests by the ATS [29]. Similarly, based on the ERS guidelines, a positive diagnosis can be made if there is presence of a hallmark ciliary ultrastructure defect on TEM plus non-ambiguous bi-allelic mutations in PCD causing genes.

## 6. Primary Ciliary Dyskinesia Diagnostic Tests

### 6.1. Genetics

There have been great advances in the PCD genetics with discovery of over 45 genes associated with PCD [5]. Approximately 70% of patients with PCD have biallelic mutations in a known gene associated with the disease [31]. These mutations are inherited in an autosomal recessive manner except for FOXJ1 (autosomal dominant) and two X-linked syndromic genes; retinitis pigmentosa (RPGR) and orofacial digital (OFD1). In all other mutations, two disease causing mutations must therefore occur in the same PCD gene to cause the disease. These genetic defects are linked to specific ultrastructural and/or functional anomalies in the ciliary axoneme [18,32]. Table 2 lists a few of the more common mutations. A comprehensive list of genes with associated ultrastructural defects can be accessed at https://www.ncbi.nlm.nih.gov/books/NBK1122/#pcd (accessed on 12 August 2020).

In patients with a clear clinical phenotype for PCD an extended genetic panel may be used as the diagnostic test in the newborn period [33]. The presence of a biallelic pathogenic mutations in a PCD gene confirms the diagnosis [29]. However, there are some important caveats. First, genetic tests often report variants of unknown significance (VUS). If the pathogenicity of the VUS in indeterminate, regular follow-up with genetics may be required to track additional information and research about the VUS as it becomes known. If the VUS is likely pathogenic, and the other allele is pathogenic, a clinical phenotype makes the diagnosis more likely. Because there can be two mutations on the same chromosome, it is often advisable to get genotypes on parents to be sure the mutations are in trans. Finally, it is important to note that single “pathogenic” recessive mutations in different genes does not make a diagnosis of PCD. It is important to have the input of a geneticist in the interpretation of these genotyping results.

### 6.2. Transmission Electron Microscopy

Up to 70% of patients with PCD have a recognizable structural defect on TEM [20]. However, diagnosis of PCD by TEM alone is no longer encouraged by the ATS. Abnormalities of cilia caused by technical difficulties of sample collection or timing of sample collection such as during a viral illness may affect ciliary structure leading to erroneous results. Additionally, adequate and consistent sample preparation as well as expertise in interpretation is required to obtain accurate results. Even when adequate samples are obtained, TEM may be inadequate in diagnosing PCD due to normal ciliary ultrastructure in certain PCD gene mutations [6]. In many cases, subtle structural abnormalities involving the central apparatus and radial spokes may be missed [7]. Lastly, common mutations that result in the absence, or reduction, of cilia may be incorrectly interpreted as an inadequate sample [34]. Taken all together, based on the ATS clinical guidelines, TEM alone cannot be used to make a conclusive diagnosis of PCD. On the contrary, the European respiratory society (ERS) recommends that in those with a hallmark ciliary ultrastructure defect for PCD, TEM alone is confirmatory for PCD and additional investigations are not required. They do however recommend that additional diagnostic work up should be performed in patients with a strong clinical history but with normal TEM.

### 6.3. Nasal Nitric Oxide

Nasal nitric oxide (nNO) is reduced in patients with PCD [35,36] and is recommended in the diagnostic panel for patients 5 years or older (ATS) or >6 years (ERS) who can successfully complete the test [29]. The testing is noninvasive, relatively inexpensive, and yields immediate results. Nasal nitric oxide can be used to screen patients with a clinical history that is suggestive of PCD followed by additional testing such as TEM or genetic tests to confirm the diagnosis. An nNO cutoff value of <77 nL/min has a >98% sensitivity and specificity for the diagnosis of PCD in individuals with a compatible clinical phenotype. Up to 30% of patients with CF may have low nNO [36]. However, after excluding CF, the diagnostic accuracy of nNO is comparable to that of TEM and/or genetic testing [37]. It is recommended that even with low nNO values clinicians should still proceed with additional confirmatory tests including genetic testing and/or TEM. This is especially important as transiently low nNO values may be seen during an acute viral infection, nasal obstruction and sinusitis and should therefore be confirmed on at least two separate occasions. The ERS guidelines however state that a very low nNO in combination with abnormal high-speed video microscopy analysis (on three occasions, or following cell culture) makes the diagnosis highly likely. A normal nNO value, however, does not rule out a diagnosis of PCD in the setting of a compatible clinical phenotype as some genetic mutations with or without classic ultrastructural defects have been associated with nNO values that are >77 nL/min [38]. The use of nNO in infants and children <5 has not been validated but its measurement using the tidal breathing technique, though less sensitive and specific, is suggested by the ERS as part of the diagnostic work up [28].

### 6.4. Assessment of Ciliary Beat Frequency and Ciliary Beat Pattern by High Speed Video Microscopy and Light Microscopy

Examination of ciliated epithelium under a microscope fitted with a high-speed video camera allows for evaluation of the ciliary beat frequency (CBF) and/or ciliary beat pattern (CBP) and has been recommended by the ERS as part of the diagnostic work-up for PCD [28]. There are, however, limitations associated with this methodology including limited expertise and equipment availability to examine cilia following regrowth from a biopsy sample. There are challenges with sample acquisition including contamination, failure to grow ciliated epithelium as well as potential functional change following regrowth due to manipulation during processing. Additionally, the inability to transfer the technology and consistent methodology across centers coupled with the lack of standards for interpretation of CBF and/or CBP poses potential for false positives and false negative results. Therefore, in patients with high probability of having PCD, the ATS suggests against using high speed video microscopy (HSVM) or light microscopy alone to assess CBF and/or CBP as PCD diagnostic tests. Additionally, ciliary waveform analysis using light microscopy without high speed is currently not recommended as a PCD diagnostic test [29].

**Figure 3:**
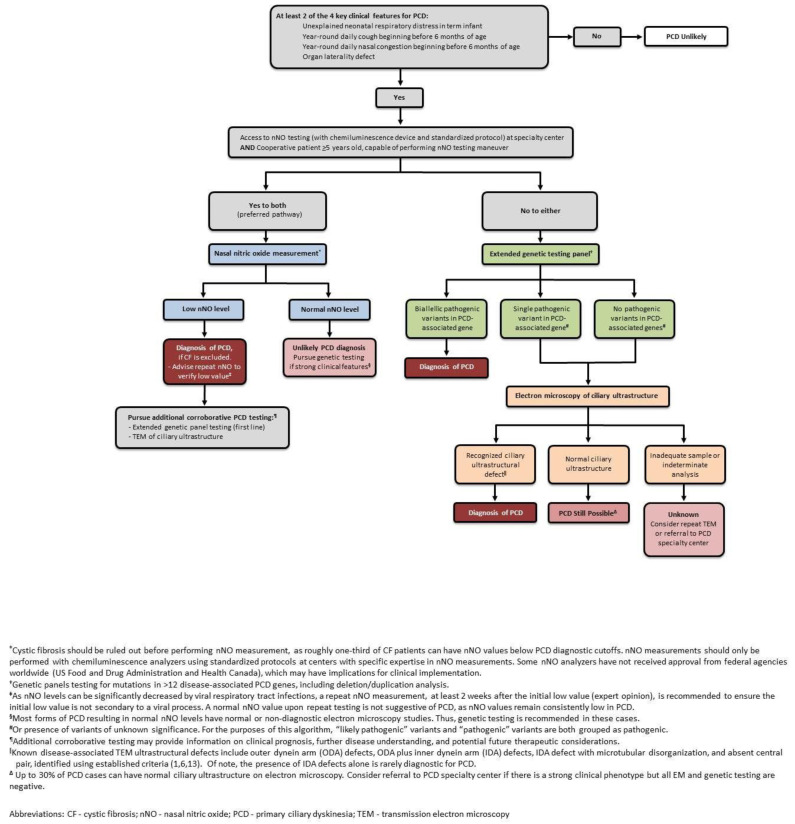
Primary Ciliary Dyskinesia Suggested Diagnostic Algorithm. Reprinted with permission of the American Thoracic Society. Copyright © 2020 American Thoracic Society. All rights reserved. Shapiro AJ, et al. Diagnosis of Primary Ciliary Dyskinesia. An Official American Thoracic Society Clinical Practice Guideline. Am J Respir Crit Care Med. 2018; 197(12): e24–e39. The American Journal of Respiratory and Critical Care Medicine is an official journal of the American Thoracic Society.

An ATS suggested diagnostic algorithm is shown in Figure 3.

## 7. Management of PCD in the Neonatal Period and Beyond

Early diagnosis and treatment of PCD-related complications could slow the progression of lung disease and avoid the development of bronchiectasis. However, there is paucity of data for recommending specific treatments in PCD. Therefore, treatment recommendations are generally centered around evidence obtained from diseases with similar pathophysiology such as CF [39] and non-CF bronchiectasis [8].

Neonates that develop respiratory distress should be aggressively managed in the neonatal intensive care unit to prevent chronic atelectasis and lobar collapse. Initial treatment strategies may include supplemental oxygen alone and/or positive pressure ventilation. Airway clearance therapy is recommended with the addition of hyperosmolar agents on a case-by-case basis. In those with refractory atelectasis and lobar collapse, flexible bronchoscopy with bronchoalveolar lavage should be considered both for diagnostic and therapeutic purposes. Respiratory cultures obtained during bronchoalveolar lavage may help direct antibiotic coverage.

Current guidelines indicate that all patients with PCD should ideally be seen in a PCD center 2–4 times a year with sputum cultures and spirometry obtained at each visit. Chest radiography should be obtained at diagnosis and every 2–4 years while computed tomography may be completed after diagnosis to detect bronchiectasis and then as needed for follow up. Daily aggressive airway clearance is recommended to mobilize the airways secretions while encouraging coughing to enhance expectoration of the mucus and maintenance of lung function [40]. Suggested airway clearance therapies include daily cardiovascular exercise, manual percussion and percussive vibratory devices including the oscillatory vest and positive expiratory pressure devices. Hyperosmolar agents such as hypertonic saline and mannitol, inhaled bronchodilators and dornase-alfa are routinely used in CF. However, these agents have not been studied in PCD, and therefore should be considered for use on a case-by-case basis [8].

As patients with PCD typically have a daily wet cough, pulmonary exacerbations are characterized by increased cough with increased mucus production, chest congestion and increased work of breathing. Chest radiography may show new areas of atelectasis or consolidation. Early administration of a broad-spectrum oral antibiotic for at least 2–3 weeks for mild exacerbations or inpatient treatment with parenteral antibiotics for severe exacerbations is recommended. The initial choice of antibiotics is generally guided by previous respiratory cultures with further adjustments based on new organism growth on sputum culture. Given lack of evidence around these practices in PCD, additional antimicrobial therapies that may be considered on case-by-case basis include inhaled antibiotics for acute exacerbations and chronic inhaled or oral suppressive antibiotics. The most common organisms isolated from the PCD airway includes Staphylococcus aureus, Hemophilus influenzae, Streptococcus pneumoniae, Moraxella catarrhalis and Pseudomonas aeruginosa. Pseudomonas aeruginosa is present both in children and adults with PCD with increasing prevalence with age [41]. The overall prevalence of both mucoid and no mucoid types is estimated to be between 20% and 36%. However, the relationship to genetic defects or ultrastructural abnormalities and the longitudinal impact on lung function is still unclear in children [27,41]. Chronic macrolide use or other anti-inflammatory medications may also be considered. In a recent multinational study chronic use of azithromycin over a period of 6 months halved the rate of pulmonary exacerbations compared to placebo [42].

Children with PCD should be evaluated 1–2 times annually by an otolaryngologist for audiologic testing and consideration for pressure equalization tubes in those with chronic middle ear effusions with hearing and/or speech impairment. There are no randomized or longitudinal clinical studies for treatment strategies for chronic rhinosinusitis (CRS) in patients with PCD. Endoscopic sinus surgery should be considered in those with severe CRS. Daily saline nasal irrigation may be beneficial for symptomatic relief, but the effects have not been studied in PCD. Acute exacerbations of CRS may be treated with antibiotics and nasal steroids.

## 8. Prognosis

Data are scanty regarding the natural progression of disease in patients with PCD and limited longitudinal observational studies have evaluated the factors influencing long term prognosis. There is great heterogeneity in the PCD clinical phenotype, yet few studies have examined the relationships and interactions between the ultrastructural defects, genetic mutations and potential environmental factors. Worse lung disease has been reported in patients with genes that result in central apparatus and microtubular disorganization, especially individuals with the CCDC39 or CCDC40 mutations [27] as well as individuals with the CCNO mutation that results in reduced number of cilia on airway epithelial cells [34]. Individuals with RSPH1 mutations typically results in normal ciliary ultrastructure on TEM [33] while those with DNAH9 mutations demonstrate impaired ciliary bending mutations [43]; these mutations typically result in milder pulmonary disease. The majority of adults develop worsening bronchiectasis and ultimately may require supplemental oxygen and/or lung transplantation. In addition, the chronic nature of the disease and the burden of daily treatments has a great impact on the quality of life affecting both the physical and emotional wellbeing across all age groups [44]. More longitudinal studies are needed to elucidate the disease progression of the >45 PCD associated genes. A recent effort establishing an international PCD database for observational studies may help answer these critical questions [45].

## 9. Conclusions

With a high index of suspicion, a diagnosis can be established for the more than 80% of individuals with PCD who present with unexplained respiratory distress, persistent oxygen requirement or organ laterality defects in the neonatal period. These findings, along with a diagnostic ciliary ultrastructure on TEM or presence of biallelic mutations in one PCD-associated gene can lead to a conclusive diagnosis. Early recognition in tern allows for treatment with aggressive airway clearance and antimicrobial therapies, which may subvert the complications associated with PCD, leading to better prognosis and improved quality of life.

## Figures and Tables

**Table 1:** Clinical manifestations associated with PCD.

Organ	Clinical Feature
Ears	Recurrent otitis media Chronic otitis media Suppurative otitis media Hearing impairment
Nose and sinuses	Early onset, year-round nasal congestion Chronic or recurrent sinusitis Nasal polyps
Lower respiratory tract	Unexplained neonatal respiratory distress Atelectasis and lobar collapse Early onset, year-round chronic cough Recurrent pneumonia Bronchiectasis
Cardiac	Heterotaxy (situs ambiguus or another organ laterality defect other than situs inversus totalis) Situs inversus totalis Congenital heart disease
Reproductive organs	Male infertility Reduced female fertility

**Table 2:** Common PCD genetic mutations with associated clinical features.

Genetic Mutation	Estimated % of PCD Pathogenic Variants	Ultrastructural Abnormality	Associated Clinical Features	Nasal Nitric Level
DNAH5	15–29%	ODA defects	Situs abnormalities	Low (<77 nL/min)
DNAI1	2–10%	ODA defects	Situs abnormalities	Low
ARMC4	<3%	ODA defects	Situs abnormalities	Low
CCDC103	<4%	ODA defects	Situs abnormalities	Low
DNAH11	6–9%	Normal	Situs abnormalities	Low
CCDC39	4–9%	IDA defects and MTD	Situs abnormalities Worse lung function	Low
CCDC40	3–4%	IDA defects and MTD	Situs abnormalities Worse lung function	Low
SPAG1	<4%	ODA+IDA defects	Situs abnormalities	Low
ZMYND10	<2–4%	ODA+IDA defects	Situs abnormalities	Low
CCNO	<2%	Oligocilia	Situs abnormalities not reported	Some > 77 nL/min

ODA: Outer dynein arm, IDA: inner dynein arm, MTD: microtubular disorganization.
